# Intranasally Delivered Adenoviral Vector Protects Chickens against Newcastle Disease Virus: Vaccine Manufacturing and Stability Assessments for Liquid and Lyophilized Formulations

**DOI:** 10.3390/vaccines12010041

**Published:** 2023-12-29

**Authors:** Omar Farnós, Barbara Cristina Martins Fernandes Paes, Belayneh Getachew, Samia Rourou, Ameni Chaabene, Esayas Gelaye, Takele A. Tefera, Amine A. Kamen

**Affiliations:** 1 Viral Vectors and Vaccines Bioprocessing Group, Department of Bioengineering, McGill University, Montreal, QC H3A 0G4, Canadabarbara.martinsfernandespaes@mcgill.ca (B.C.M.F.P.); 2 Research and Development Directorate, National Veterinary Institute, Bishoftu P.O. Box 19, Ethiopiaesayasgelaye@gmail.com (E.G.); takele.abayneh@nvi.com.et (T.A.T.); 3 Laboratory of Molecular Microbiology, Vaccinology and Biotechnology Development, Group of Biotechnology Development, Institut Pasteur de Tunis, Université Tunis El Manar, 13, Place Pasteur. BP.74., Tunis 1002, Tunisia; samia.rourou@pasteur.tn (S.R.);

**Keywords:** newcastle disease virus, adenovirus vaccine, vaccine manufacturing, intranasal vaccination, mucosal protection, HEK293 suspension cells, bioreactor production, downstream processing, veterinary vaccine production platform

## Abstract

Newcastle disease (ND) remains a critical disease affecting poultry in sub-Saharan Africa. In some countries, repeated outbreaks have a major impact on local economies and food security. Recently, we developed an adenovirus-vectored vaccine encoding the Fusion protein from an Ethiopian isolate of Newcastle disease virus (NDV). The adenoviral vector was designed, and a manufacturing process was developed in the context of the Livestock Vaccine Innovation Fund initiative funded by the International Development Research Centre (IDRC) of Canada. The industrially relevant recombinant vaccine technology platform is being transferred to the National Veterinary Institute (Ethiopia) for veterinary applications. Here, a manufacturing process using HEK293SF suspension cells cultured in stirred-tank bioreactors for the vaccine production is proposed. Taking into consideration supply chain limitations, options for serum-free media selection were evaluated. A streamlined downstream process including a filtration, an ultrafiltration, and a concentration step was developed. With high volumetric yields (infectious titers up to 5 × 10^9^ TCID50/mL) in the culture supernatant, the final formulations were prepared at 10^10^ TCID50/mL, either in liquid or lyophilized forms. The liquid formulation was suitable and safe for mucosal vaccination and was stable for 1 week at 37 °C. Both the liquid and lyophilized formulations were stable after 6 months of storage at 4 °C. We demonstrate that the instillation of the adenoviral vector through the nasal cavity can confer protection to chickens against a lethal challenge with NDV. Overall, a manufacturing process for the adenovirus-vectored vaccine was developed, and protective doses were determined using a convenient route of delivery. Formulation and storage conditions were established, and quality control protocols were implemented.

## 1. Introduction

Newcastle disease (ND) remains one of the most prevailing infectious diseases with a negative impact on poultry farming in sub-Saharan Africa. It economically impacts producers at different scales and causes the deterioration of food security in the region [1,2,3,4]. In sub-Saharan Africa, chickens are also farmed in small villages and rural settings by women, which are the main work force in the agricultural sector. In these settings, ND can have devastating effects for small-scale producers, affecting their economic independence [5,6]. Immunizing poultry with inactivated or live-attenuated ND vaccines is the current prevention method; however, there are critical limitations with the supply chain of pathogen-free embryonated eggs and other disadvantages, such as possible virus shedding [7]. The present manufacturing process of NDV vaccines in Africa is costly. It involves pathogen-free chicken embryonated eggs which are imported from countries outside the continent [5,8,9,10]. Additionally, most available vaccines are not genotype-specific and are being prepared using Newcastle disease virus (NDV) strains isolated many years ago in geographically distant locations. Cell culture technologies are a valid alternative for more effective vaccine production processes. Such technologies can also be adapted to other pathogens in case of newly emerging outbreaks.

Considering the economic impact of ND infections and the current production methods for NDV vaccine production, we recently developed a streamlined and efficient technology platform that uses the human adenovirus serotype 5 and HEK293SF cells to produce a virus-vectored vaccine against NDV infection. The adenovirus encodes the Fusion antigen from a recent local isolate of NDV from Ethiopia [11]. It was produced in stirred-tank bioreactors, purified by ultracentrifugation, and delivered through intramuscular injection for the immunization of chickens, which were fully protected against a lethal challenge with NDV. This viral vector was selected due to its extensive use in vaccination (including against COVID-19 for human populations), its “self-adjuvanting” properties, and its well-controlled and robust manufacturing process [12,13,14]. The system has a demonstrated safety; it supports high viral yields at relatively low costs and is of easy implementation for a veterinary product [15,16,17,18,19,20].

In this work, we present a further improved upstream process and develop a new downstream process for manufacturing the adenovirus-vectored vaccine. This resulted in a clarified product that was obtained mainly through filtration and ultrafiltration steps, being formulated in a liquid formulation that demonstrated remarkable stability. Stability assessments included thermally stressed storage conditions or long-term storage under standard conditions. Additionally, new formulations to sustain a lyophilization process were developed and further optimized. For the first time, mucosal immunization through the nasal cavity of chickens with the adenoviral vector encoding the F antigen from NDV was evaluated in one immunization and challenge experiment, in which different doses were assessed. Protection against a heterologous challenge was also evaluated.

## 2. Materials and Methods

### 2.1. Cells Lines and Culture Media

The Median Tissue Culture Infectious Dose (TCID50) assay was conducted using HEK293A adherent cells. HEK293A cells were cultured in dishes in a humidified incubator at 5% CO_2_ and 37 °C in Dulbecco’s Modified Eagles Medium (DMEM) (Wisent, Quebec, QC, Canada), which was supplemented with 10% Fetal Bovine Serum (FBS) (Gibco, Grand Island, NY, USA). Passage of the cells was performed twice a week with detachment at confluence with Trypsin (Millipore Sigma, Oakville, ON, Canada). Cells were centrifuged at 400× *g* for 5 min, resuspended in fresh medium, and seeded at ten times dilution.

The HEK293SF cell line (clone 293SF-3F6) is derived from HEK293 cells adapted to grow in suspension and serum-free medium. These suspension cells were derived from a GMP master cell bank [21,22]. These cells were used for adenoviral vector production in bioreactors at a scale of 1 or 3 L. HEK293SF cells were grown in disposable polycarbonate vented cap shake flasks (TriForest Enterprises, Irvine, CA, USA) and stirred-tank bioreactors. Cells were routinely passaged twice per week by diluting to 2.5 × 10^5^ viable cells per mL in fresh medium. Two media were used to grow the cells: HyClone HyCell TransFx-H medium (Cytiva, Logan, UT, USA) and Xell HEK-GM medium (Sartorius Xell, Bielefeld, Germany). Both are chemically defined, animal-component-free, and protein-free media, with no antibiotics. HyCell TransFx-H was supplemented with 4–6 mM Glutamax (Gibco, Grand Island, NY, USA) and 0.1% Kolliphor poloxamer 188 (Millipore Sigma, Oakville, ON, Canada). When grown in HEK-GM, it was supplemented with 4–6 mM GlutaMAX™ (Gibco, Grand Island, NY, USA). Cell growth and viability were determined using trypan blue in a Vi-CELL-XR Cell Viability Analyzer (Beckman Coulter, Mississauga, ON, Canada).

### 2.2. Cell Growth and Virus Production at Small Scale

Cell growth of HEK293SF cells was conducted in batch and fed-batch mode in HycellTransFx-H (Cytiva, Logan, UT, USA) and HEK-GM (Sartorius Xell, Bielefeld, Germany), using shake flasks (TriForest Enterprises, Irvine, CA, USA) in an orbital shaker platform (Infor’s HT, Montréal, QC, Canada) at 135 revolutions per minute (rpm), with 75% humidity, 5% CO_2_, and temperature of 37 °C. Culture seeding was conducted at a cell density of 0.25 × 10^6^ cells/mL. For fed-batch cultures, the addition of supplements consisted, in the case of HEK-GM basal media, of bolus addition of HEK-FS (Sartorius Xell, Germany) supplemented with 4 mM GlutaMAX™ (Gibco, Grand Island, NY, USA), at 3% (*v*/*v*), at 24 h post-seeding and then every 48 h until harvest. Supplementing the HyCellTransFx medium was conducted with Cell Boost 5 (Cytiva, Logan, UT, USA), using the same schedule, adding a bolus at 5% (*v*/*v*) supplemented with 4 mM GlutaMAX™ (Gibco, Grand Island, NY, USA) until the end of the production phase. Two approaches were followed: (i) supplementing until the time of infection and (ii) supplementing during the complete process. Cell counts were performed daily. For infection and virus production characterization, HEK293SF cells were cultured until they reached densities of 2 × 10^6^ cells/mL. Infections were performed at MOI equal to 0.35. During production, cells were monitored for cell density and viability daily. For every shake flask analyzed, at 72 hpi, the harvest was performed, samples were collected, and the cells were lysed with three freeze/thaw cycles, alternating between incubations at −80 °C and 30 °C in a water bath. After cell lysis, DNase treatment with 5 U/mL of Benzonase (Millipore, Oakville, ON, Canada) was performed for 1 h at 37 °C. The lysate was centrifuged at 6000× *g* for 15 min at 4 °C. Alternatively, at harvest, the cells were lysed with lysis buffer (10 mM HEPES, 0.5% *w/v* Tween-20, 2 mM MgCl_2_) for 1 h under agitation, followed by the DNase treatment. The cell culture lysate containing the virus was quantified via TCID50 assay for infectious particle concentration. Measurements were conducted in duplicate.

### 2.3. Adenovirus Production in Bioreactors

Adenoviral production in 3 L bioreactors (Applikon Biotechnologies, Delft, The Netherlands) was conducted in batch or fed-batch mode as previously described for shake flasks, with the Ad-CMV-F adenoviral vector. The 3 L bioreactor was equipped with a pH sensor and a dissolved oxygen (DO) concentration sensor. The bioreactor (working volume 2.7 L) was also equipped with a double marine impeller and a capacitance probe (Aber Instruments Ltd., Aberystwyth, UK). The reactors were seeded at a viable cell density around 0.35 × 10^6^ cells/mL in HyCellTransFx-H medium, and cell growth was allowed until the time of infection at approximately 2 × 10^6^ cells/mL and MOI up to 3.5. The culture runs were supplemented with 5% (*v*/*v*) Cell Boost 5 and 1 mM L-Glutamine. The bioreactor ensured controlled conditions with a DO concentration at 40% air saturation by continuous surface aeration of 5 to 12.5 mL/min air (for the 1 or 3 L units, respectively) and injection of pure oxygen through the sparger when required. The pH was set to 7.15 and regulated by the injection of CO_2_ or the addition of NaHCO_3_ (90 g/L) (Sigma, Burlington, MA, USA). Agitation was kept maintained at 90 rpm and increased to 120 rpm in the last 24 h of culture. Monitoring of cell growth and infection process was also followed through the analysis of capacitance values (uF/cm). The bioreactor control unit ensured controlled conditions of the process, via the proportional–integral–derivative (PID) controller. Cells were harvested when cell viability reached around 60–75%, at about 44–94 hpi. TCID50/mL values were also determined.

### 2.4. Downstream Processing

#### 2.4.1. Cell Harvest and Lysis

To harvest the bioreactor production, the cells were either collected, centrifuged at 1000× *g* for 15 min, and lysed with three freeze/thaw cycles, alternating between incubations at −80 °C and 30 °C, or were instead chemically lysed inside the bioreactor with 10 mM HEPES, 0.5% *w*/*v* Tween-20, and 2 mM MgCl_2_ buffer. After cell lysis, in both cases, DNase treatment was performed with 5 to 10 U/mL of Benzonase (Millipore, Oakville, ON, Canada) for 1 h at 37 °C. Two approaches were followed for separation from the cell debris of lysed cells; the lysate was either centrifuged at 6000× *g* for 15 min at 4 °C to remove cell debris or subjected to a depth filtration step (no centrifugation) for clarification and separation from the cell debris generated. The cell culture lysate supernatant containing the virus was quantified via TCID50 assay for infectious particle concentration.

#### 2.4.2. Purification by CsCl Gradient Ultracentrifugation or Clarification through Depth Filtration and Tangential Flow Filtration Steps

For purification in CsCl density gradients, cell lysate supernatants were loaded onto a two-step CsCl gradient and ultracentrifuged at 28,500 rpm (100,000× *g*) at 4 °C for 90 min. The virus band was collected with tube puncturing. Dialysis was immediately carried out using the Slide-A-Lyzer G2 Dialysis Cassettes (300 kDa cut-off) against the formulation buffer, as described by Farnós et al. [11].

**Figure 1:**
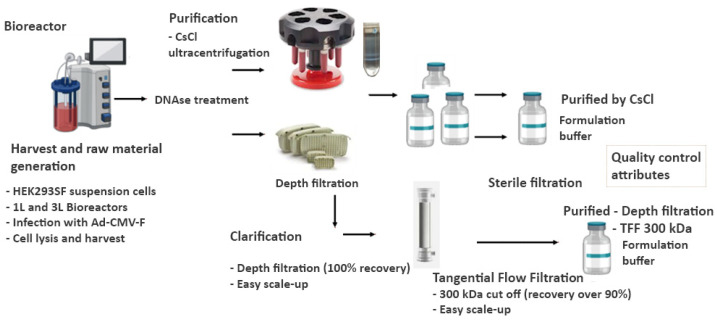
Downstream processes for the purification or clarification of the Ad-CMV-F adenoviral vector. Part of the production was subjected to ultracentrifugation in CsCl gradients for purification of the virus, while the other part was taken for a clarification streamlined process that involved a depth filtration step followed by a tangential flow filtration for ultrafiltration and 10–20× concentration purposes.

For a simplified clarification process, the cell culture lysate-containing virus was filtered using the Millistak+^®^ Depth Filter in µPod^®^ format (D0HC media series, 23 cm^2^ surface area) from Merck KGaA (Darmstadt, Germany) to remove the cell debris. Then, a tangential flow filtration step with hollow-fibre cartridges MIDIKROS 20 cm 300 kDa and 500 KDa from Repligen (Rancho Dominguez, CA, USA) was performed to exchange the buffer and concentrate the product in the desired formulation buffer. For smaller volumes after TFF, an additional Amicon concentration step was used (Figure 1).

### 2.5. Assessment of Product Quality

#### 2.5.1. Analytical Assays for Characterization of the Recombinant Adenovirus

##### Total Particle Quantification of the Adenoviral Vector

For quantification via Digital Droplet Polymerase Chain Reaction (ddPCR): Viral DNA extraction was conducted for 20 µL of supernatant samples diluted with 180 µL PBS. The High Pure Viral Nucleic Acid kit (Roche, Basel, Switzerland) was used for extraction. The DNA was diluted (dilutions between 1:10 and 1:10,000). In total, 5 µL of the template dilution was used with the QX200 ddPCR kit (Bio-Rad Laboratories, Hercules, CA, USA), using the EvaGreen master mix and primers to amplify the Fusion gene; Fw: TTAGCTGGTGGCAATATGGA and Rv: TCATGTCCTTGTAGTAGCTCTCATC. The program was the following: initial denaturation (5 min at 95 °C), 34 cycles of the following steps: 30 s at 95 °C, 1 min at 59 °C, and 30 s at 72 °C. A final elongation step was conducted for 5 min at 72 °C.

##### Titration of Infectious Particles

Infectious titers (IVP/mL) were determined using HEK293A and 96-well plates. Cells were seeded at 20,000 cells/well in DMEM supplemented with 2% FBS and allowed to grow for 24 h. Then, serial dilutions of the virus samples were prepared in fresh medium that was added to the plate in duplicates, with two plates per sample (16 wells per sample). Starting dilutions were 10^−3^ or 10^−4^ depending on the expected titer. Subsequent 1:10 dilutions were performed horizontally in the plate, keeping one column as negative control with no virus presence. After 10–14 days, the cells were visualized using a microscope for the development of the cytopathic effect. The TCID50/mL value was calculated according to Reed and Muench [23]. The average coefficient of variation (CV) was also calculated for the TCID50 assay, resulting in ±27%. The average CV is the arithmetic mean of the coefficient of variation from the 7 independent runs, each with two or three replicates.

#### 2.5.2. Host Cell Protein and Total DNA Determinations

Host cell protein content (HCP) was determined in the final step of the clarification/purification process using the HEK293 HCP ELISA Kit from Cygnus (Leland, NC, USA) to monitor the remaining host cell impurities in the products.

Total DNA quantifications were examined using the Quant–iT Picogreen dsDNA Determination Assay from Invitrogen (Eugene, OR, USA).

### 2.6. Liquid and Solid Formulations for Ad-CMV-F Vaccine Storage

#### 2.6.1. Reagents and Experimental Design

Formulations for storage of Ad-CMV-F in liquid form and solid form were prepared and subjected to sterile filtration. After a series of preliminary assessments involving many different additives, the following excipients and reagents were included in the formulations for the stability assessments: Sucrose, magnesium chloride, Kolliphor P188, and polysorbate-80 were purchased from Sigma-Aldrich (St. Louis, MO, USA). Sodium chloride and tris (hydroxymethy1)-aminomethane were purchased from BioShop (Burlington, ON, Canada). Trehalose and sorbitol were purchased from Fisher Bioreagents (Fair Lawn, NJ, USA). Inulin was obtained from ThermoFisher (Brussels, Belgium). Histidine was obtained from VWR (Mississauga, ON, Canada). Ethanol was obtained from Alcools de Commerce (Boucherville, QC, Canada). D-mannitol was obtained from BioBasic (Markham, ON, Canada).

For the liquid formulations, the concentrated Ad-CMV-F stock was thawed and mixed with excipient solutions, as shown in Table 1. Briefly, for the 5 liquid formulations, the adenoviral vector (at 5 × 10^9^ TCID50/mL) was thawed, and a buffer exchange step was performed using the centrifugal filter unit Amicon 100 kDa (MilliporeSigma™, Darmstadt, Germany). The final virus concentration ranged from 2 × 10^9^ to 5 × 10^9^ TCID50/mL. Then, aliquots of 100 µL were transferred into 1.5 mL vials and stored at different temperatures. The five formulated products in liquid form were stored at room temperature (21.5 °C), 37 °C, 4 °C, and −80 °C for 1, 2, 4, 16, and 24 weeks for the stability study. A similar process was conducted for the sample preparation with the solid formulations (Table 2). Briefly, for the 3 solid formulations, the adenoviral vector (3 × 10^10^ TCID50/mL) was thawed and diluted with sterilized excipient solutions into the desired solid formulation. The final virus concentration ranged from 7 × 10^7^ to 3 × 10^8^ TCID50/mL. Then, aliquots of 1 mL were transferred into 2 mL glass vials for lyophilization and stored at different temperatures. The formulated products in solid form were stored at 21.5 °C, 37 °C, and 4 °C for 1, 4, and 24 weeks for the stability assessment. After storage at the indicated temperature and the time mentioned, the Ad-CMV-F-vectored vaccine was quantified in liquid and solid forms by TCID50 assays.

#### 2.6.2. Lyophilization

Lyophilization was performed using an SP Virtis Advantage Pro Freeze Dryer (SP Scientific, Warminster, PA, USA). One milliliter of each formulated product containing the Ad-CMV-F was filled into glass vials and partially stoppered. Cycle parameters were extensively studied and optimized and are tabulated in the Results section. These parameters were modified from those recently reported [24,25], with the addition of an annealing step with a temperature of −15 °C for 2 h during the freezing step, an extension of the primary drying phase to 35 h at −50 °C, and shortening of the tertiary drying phase to 15 h at 20 °C.

The lyophilized vaccine was reconstituted for infectivity titration with sterile ultrapure water and vortexed for 5 s. Residual moisture was calculated after the lyophilized samples were kept for 7 h in the oven at 100 °C for complete drying. Calculations were conducted as follows:

$$Residual\;moisture\left(\%\right)=\frac{\left(Weight\;after\;lyophilization-weight\;after\;complete\;drying\right)}{\left(weight\;after\;complete\;drying-weight\;of\;empty\;vials\right)}\times 100$$

### 2.7. Immunization Experiments and Viral Challenge

#### 2.7.1. Ethics Statement

The experimentation with animals was in accordance with recommendations from the Guide for the Care and Use of Laboratory Animals and internal policies from the National Veterinary Institute, Ethiopia. Chickens were housed in appropriate rooms, supplied with feeding, water, and health monitoring. The experimental protocols were approved by the Institutional Committee of Ethics.

#### 2.7.2. Vaccination and Challenge with NDV

The NDV isolate NDV/Debre zeit/2018 [MN909678] was employed to administer an intramuscular homologous challenge to chickens, at a lethal dose of 0.5 × 10^6.5^ ELD50.

Live thermostable vaccine from the NDV I2 strain was prepared at the National Veterinary Institute, Ethiopia, and used as positive control of vaccination.

A heterologous Newcastle disease virus isolate was used for the administration of an intramuscular heterologous challenge to chickens, using a dose of 0.5 × 10^6.5^ ELD50.

#### 2.7.3. Immunization Experiment in Chickens

Different lots from two productions in 3 L bioreactors were used for the immunization of chickens. CsCl-purified lots and Ad-CMV-F purified by depth filtration and TFF steps were evaluated in immunizations by parenteral and mucosal routes. One- to two-week-old male and female chickens were selected at random and distributed into 10 groups, each one with 10 chickens, which were reared in-house at NVI. Three groups of chickens were parenterally immunized. The other five groups of the animals were mucosally vaccinated by intranasal instillation of the liquid formulation in one nostril, using a pipette as the dispenser. Two of the three groups, parenterally vaccinated, received a dose of approximately 1 × 10^9^ TCID50 (measured as 2.4 × 10^11^ Vg/mL) of the recombinant Ad-CMV-F purified by CsCl, formulated as previously described. These groups were immunized by i.m. and s.c. routes, with 100 µL total volume. A third group was intramuscularly vaccinated with a dose of CsCl-purified adenovector at 1 × 10^8^ TCID50 and challenged with a heterologous NDV strain. Five other groups were intranasally immunized by drop instillation into the nasal cavity with 100 or 200 µL of the Ad-CMV-F vaccine. When the instillation volume was 200 µL, it was administered as 100 µL per nostril, in accordance with previous studies and routine practices with poultry at NVI. Three of the i.n. immunized animals received the Ad-CMV-F vector in purified form and liquid formulation. The doses administered were 1 × 10^9^, 1 × 10^8^, and 0.5 × 10^7^ TCID50. The two other groups received the Ad-CMV-F formulations obtained after clarification of the culture supernatant via depth filtration and TFF, as described in Section 2.4.2, with doses of 1 × 10^9^ and 5 × 10^8^ TCID50. Finally, one positive control group was vaccinated with eye drops using the NDV live-attenuated vaccine, following the manufacturer’s indications on dose and schedule, while a tenth group remained unvaccinated. The immunizations were administered 21 days apart, and the viral challenge was practiced on day 56 of the trial with the NDV isolate Debre zeit/2018 at a lethal dose of 0.5 × 10^6.5^ ELD50, intranasally administered. All vaccination and challenge experiments were conducted at the National Veterinary Institute, Ethiopia.

## 3. Results

### 3.1. Upstream and Downstream Processing of the Ad-CMV-F Adenovirus-Based Vaccine

#### 3.1.1. Process Media and Additives for Adenovirus Production

The adenoviral vector carrying the F gene from a circulating NDV Ethiopian isolate (Ad-CMV-F) was produced in shake flasks and 3 L bioreactors, following batch and fed-batch cultivation modes with two different media, as explained in the Materials and Methods. After a series of preliminary experiments, it was shown that supplementing any of the two culture media assayed at the concentrations described provided advantages in terms of increased infectious titers measured in the culture supernatant after cell lysis. The addition of supplements to the culture during the cell growth and production phases resulted in the highest titers, in the range of 10^9^ TCID50/mL (Table 3). Different fed-batch productions in shake flasks and 3 L bioreactors, using HyCell TransFx-H medium and MOI up to 3.5, were run and are summarized in Table 4. These productions were achieved to support downstream processing studies, stability and formulation studies, and the production of material for immunization trials in chicken. These runs yielded maximum titers of up to 5.10 × 10^9^ TCID50/mL before any processing or concentration steps.

#### 3.1.2. Downstream Processing

After harvest, chemical cell lysis, and DNase treatment with Benzonase, two different methodologies were followed for the processing and separation of the adenoviral vector from the cell lysate supernatant. The ultracentrifugation in CsCl gradients was previously described [11]. It produces a highly concentrated and purified virus that demonstrated its protective capacity in chickens administered by a parenteral route. The second approach, as an alternative downstream method to ultracentrifugation, consisted of clarification of the cell lysate supernatant via depth filtration, followed by a tangential flow filtration step for a 10–20-fold concentration and buffer exchange. The depth filtration step with the DØCH membrane, which avoids the need for high-speed centrifugation for separation of cell debris, rendered recoveries of 100% of total and infectious particles after being repeatedly evaluated with different buffers to reach an efficient cell lysis. After this first clarification step and concentration with the tangential flow filtration (TFF) set-up, infectious viral particles with functional titers of up to 5.62 × 10^9^ TCID50/mL were measured.

When comparing the filtration performed with the 300 kDa and the 500 kDa cut-off columns, 100% recovery was achieved with the 300 kDa column compared to 27.83% recovery with the 500 kDa column (*n* = 1). An excellent recovery capacity was seen for the TFF step, in which virus loss in the permeate fraction was quantified, and it was below 0.0053% in the case of the 300 kDa hollow fibre. The final product was always concentrated around 10–20 fold, subjected to sterile filtration, and quantified de novo. Table 5 shows different measurements at the depth filtration and TFFs steps, showing the step recovery levels in different fraction tests from the bioreactor runs. After the clarification and sterile filtration steps, maximum volumetric yields of up to 10^12^ IVP per liter of culture were measured. Table 6 summarizes the virus titers for the two productions processed which were further used for poultry vaccination.

In addition to measurements of infectious and total viral particles, the analysis on the content of contaminant DNA and host cell protein levels was performed as quality control on lots prepared by CsCl gradients or by depth filtration followed by tangential flow filtration. In the case of the clarified adenovirus, samples filtered by the 300 kDa or the 500 kDa hollow-fibre cartridges were evaluated. The results showed lower amounts of DNA (in the range of 0.3–0.4 µg/mL) in the samples from clarified formulations (both 300 kDa and 500 kDa) compared to those subjected to ultracentrifugation (around 1.2–1.3 µg/mL), most likely due to variations in cell density at the time of harvest. The host cell protein levels showed a concentration of around 20 µg/mL in all the samples subjected to clarification, while the Ad-CMV-F samples purified by CsCl exhibited levels of around 0.2–0.3 µg per mL.

### 3.2. Stability of the Ad-CMV-F Adenoviral Vector

#### 3.2.1. Stability Study for Storage Using Liquid Formulations

**Figure 2:**
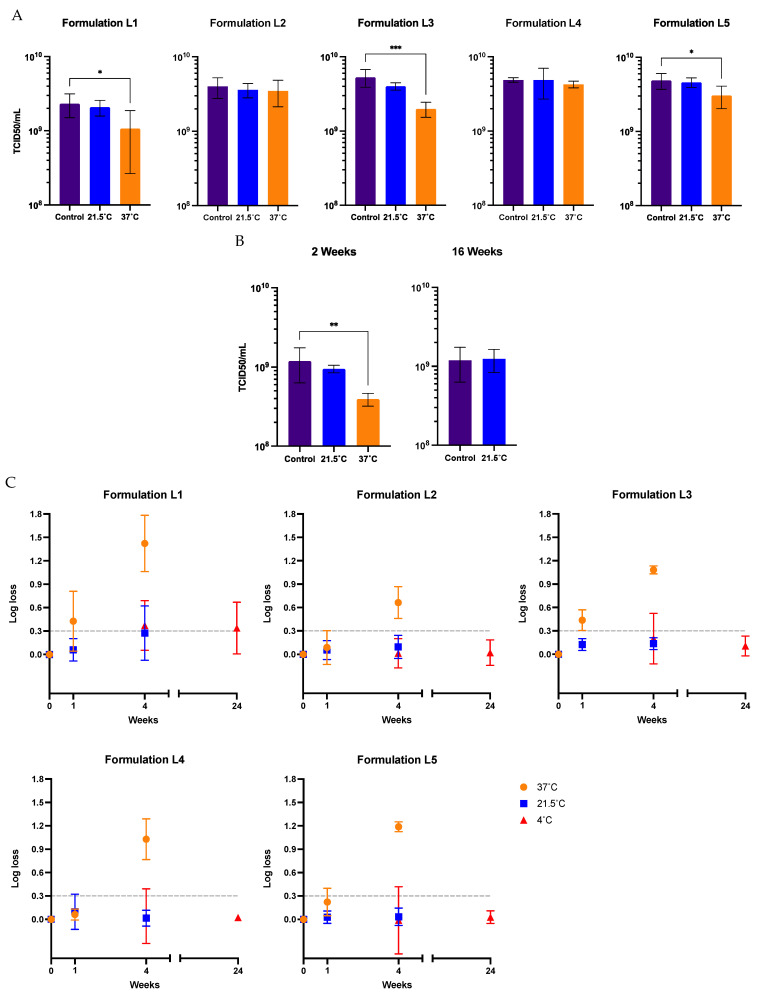
Functional titer loss after storage at 37 °C, 21.5 °C, and 4 °C in five different liquid formulations, as compared to the initial titer before treatments (designated as the control in panels (**A**,**B**)). (**A**) Virus stored for 1 week at 21.5 °C and 37 °C. (**B**) Virus stored in formulation 2 for 2 weeks at 37 °C and 21.5 °C and 16 weeks at 21.5 °C. (**C**) Storage up to 24 weeks. Values represent mean ± standard deviation (*n* = 5), *** *p* < 0.001, ** *p* < 0.01, * *p* < 0.05, by analysis of variance (ANOVA) followed by a Dunnett’s post test. A Student’s *t*-test was also conducted, depending on the number of groups under analysis. The dashed line represents the threshold for a 50% loss in titer, equivalent to 0.301 log. Formulations L2–L5 maintained identical values of infectivity after storage at −80 °C.

The Ad-CMV-F adenoviral vector was subjected to different temperature stress treatments and storage conditions to evaluate the capacity of the final formulations under study to withstand potential adverse conditions during field applications. Adenoviral sample formulations were treated and quantified after storage at 37 °C, 21.5 °C, 4 °C, and −80 °C for 1, 4, and 24 weeks in five liquid formulations. The formulations assayed (see the Materials and Methods) contained different key excipients with varied potential to provide long-term stability to the adenoviral vector. Notably, the titer was maintained after 1 week at 21.5 °C across all the liquid formulations. However, after 1 week at 37 °C, a significant functional titer loss was observed for formulations L1 and L3, resulting in a loss of infectivity of more than 54%. Formulation L5 had an average loss of 37.3 ± 21.1% after 1 week at 37 °C (Figure 2A).

The formulation L2 maintained the virus activity after 1 week at 37 °C with a minor 12.8 ± 33.8% loss of infectivity, which falls within the inherent assay variability of ±27%, calculated as described in the Materials and Methods. From a linear regression, a half-life at 37 °C was estimated to be 2 weeks. In line with this estimation, the experimental results for L2 demonstrated an average loss of 66.9% ± 6.1% of infectivity after 14 days at 37 °C (Figure 2B).

**Figure 3:**
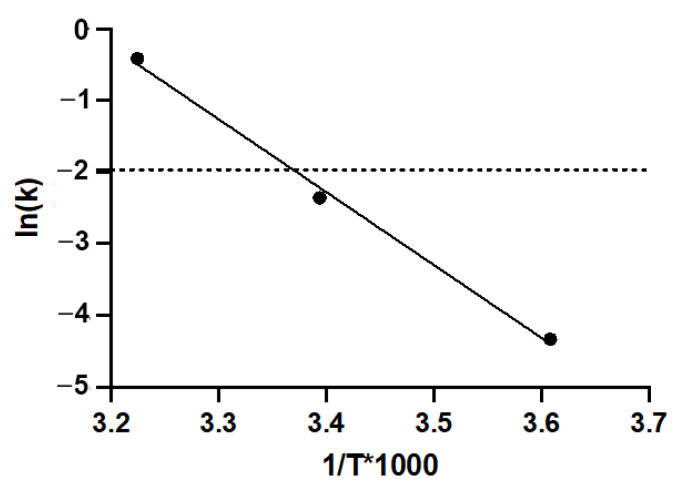
Arrhenius plot of Ad-CMV-F stability data in formulation L2. The *y*-axis shows the natural log (LN) of the infectivity loss (k), where k is expressed as log loss (base 10). The data are shown as the mean. T = absolute temperature in Kelvins. The dashed line represents a titer loss of 0.14 log (i.e., 27%), equivalent to the average variability in the TCID50 assay.

A prolonged storage time was also analyzed. A loss in titer of 50% is represented by the dashed line on the 0.301 log mark on the *y*-axis of Figure 2C. After 4 weeks at 37 °C, a significant loss in titer, above 78.3%, was observed for all the formulations. After 4 weeks at 21.5 °C, the functional titer decreased significantly in formulation L1, above 46.8% loss in titer. However, the infectious titer was maintained in the other four formulations at this temperature. Additionally, formulation L2 demonstrated, after 16 weeks at 21.5 °C, its ability to preserve the virus titer (Figure 2B). Using an Arrhenius plot, it was estimated that the Ad-CMV-F stored for 4 weeks in formulation L2 at temperatures ≥23.6 °C will have a functional titer loss above 0.14 log (represented by the dashed line on −1.96 ln mark on the *y*-axis of Figure 3), indicating a significant loss due to degradation rather than assay variability. Infectivity loss (k) was determined from Figure 2B, specifically at the 4-week storage time point at 4 °C, 21.5 °C, and 37 °C.

At 4 °C, formulations L2 to L5 maintained the titer after 4 and 24 weeks of storage. The formulation L1 lost 57.5% of its functional titer after 4 weeks (Figure 2C). A linear regression analysis of the data in Figure 3B showed that the infectivity loss rate at 4 °C for formulation L2 is 0.0031 log per month. Notably, the infectivity loss after 24 weeks of storage at 4 °C remained ≤0.10 log. These data indicate that the half-life (t1/2) for Ad-CMV-F infectivity loss in L2 at 4 °C is 94.7 months, i.e., 7.9 years. Furthermore, among all the formulations, only formulation L1 exhibited a loss in infectivity after storage at −80 °C for 24 weeks, with a loss of 0.32 log (equivalent to a 52.6 ± 15.3% reduction in titer).

#### 3.2.2. Stability Study for Adenovirus Storage using Solid Formulations

##### Characterization and Optimization of the Lyophilization Cycles

**Figure 4:**
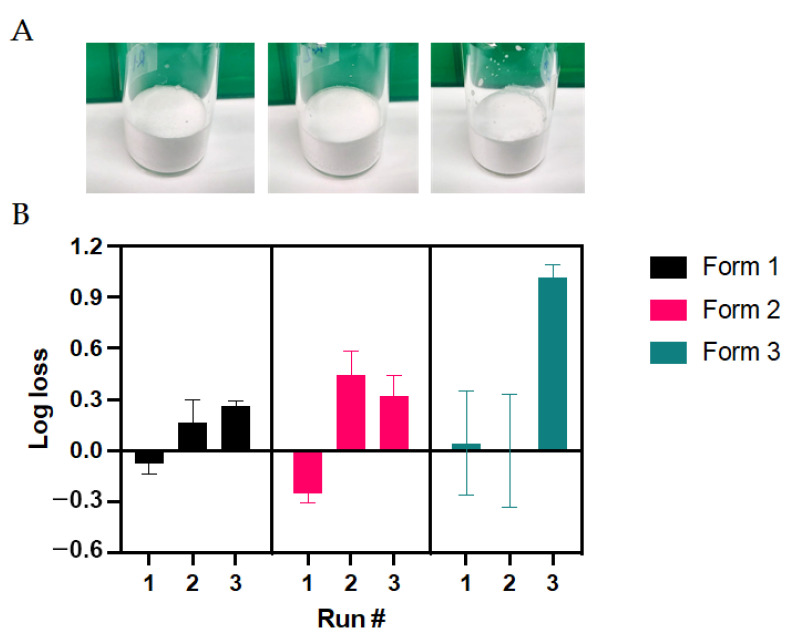
Characterization of the three solid formulations after lyophilization cycle optimization. (**A**) Representative images depicting cake formation in the three solid formulations. (**B**) Functional titer loss after lyophilization in three different runs. Values represent mean ± standard deviation (*n* = 2 for runs #1 and #3; n = 4 for run #2).

The formulations evaluated for the lyophilization process were described in the Materials and Methods. Three solid formulations varying solely in their cryoprotectant component, sucrose (S1), trehalose (S2), and inulin (S3), were evaluated. The optimized cycles for the lyophilization of the Ad-CMV-F are detailed in Table 7. These cycles were reached after several series of tests for optimization, with assessments of the physical properties of the dried samples and their infectious titer after the process. With these cycles, cakes with satisfactory visual characteristics for the three proposed formulations were generated (Figure 4A). The residual moisture was quantified in samples from the three solid formulations in two separate experimental runs. In the case of the solid formulation S1, the residual moisture content post-freeze-drying was determined to be 4.74 ± 0.94%. For solid formulation S2, this value was measured at 5.66 ± 0.71%, while for solid formulation S3, it was 3.63 ± 1.64%. Notably, all these levels fell within the satisfactory range of 3 to 6% of residual moisture, indicative of a successful lyophilization process.

The reproducibility of the cycle was evaluated from three independent runs. For each experimental run, the Ad-CMV-F vector was quantified before and after the lyophilization process to measure any detrimental effect intrinsic to the process on the infectious titer. For the solid formulation S1, the average log loss was 0.11 log, representing around 23.4% loss in titer. This decrease falls within the expected assay variability. However, solid formulations S2 and S3 showed an average titer loss of 32.4% and 55.5%, respectively (Figure 4B).

**Figure 5:**
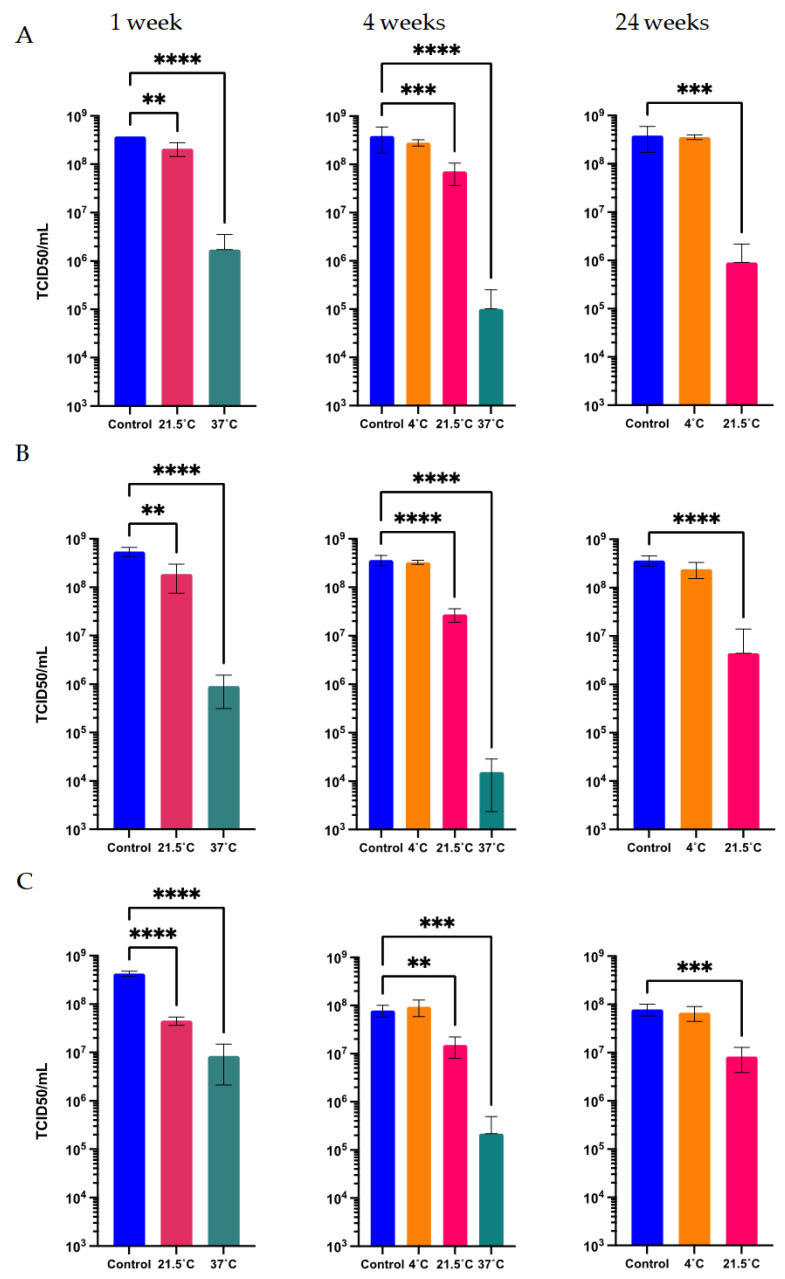
Functional titer after storage at 37 °C, 21.5 °C, and 4 °C for 1, 4, and 24 weeks in different solid formulations. The control bar represents the result of infectious titer before the different storage treatments were initiated. (**A**) Solid formulation S1. (**B**) Solid formulation S2. (**C**) Solid formulation S3. Values represent mean ± standard deviations (*n* = 5), **** *p* < 0.0001, *** *p* < 0.001, ** *p* < 0.01, according to an analysis of variance (ANOVA) followed by the Dunnett’s test. All formulations maintained equivalent infectivity levels while stored at −80 °C.

The stability of the three solid formulations during prolonged storage was analyzed by quantifying the Ad-CMV-F vector after 1, 4, and 24 weeks of storage at different temperatures and comparing it to a control before lyophilization (i.e., considering in-process and in-storage losses) (Figure 5). After 1 week at 21.5 °C, all formulations presented a significant functional titer loss, with storage in solid formulation S1 resulting in the lowest loss compared to the other formulations (around 43.6 ± 17.9%) (Figure 5A). Significant titer loss was observed for the three solid formulations after 1 and 4 weeks at 37 °C. Nonetheless, after extended storage at 4 °C, the three solid formulations were able to maintain the titer levels, demonstrating a significant capacity for preservation even over the course of 24 weeks.

**Figure 6:**
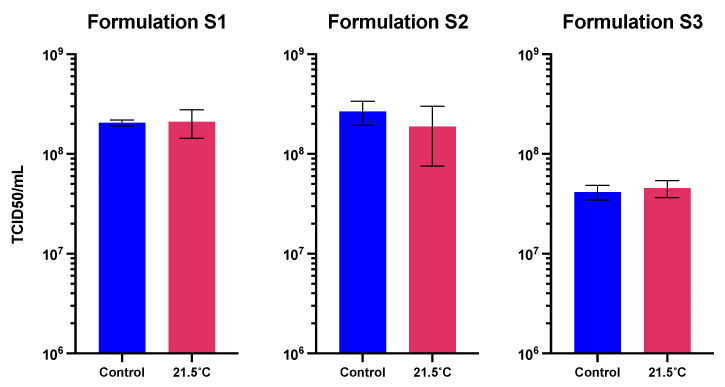
Functional titer after storage at 21.5 °C for 1 week in the three solid formulations, compared to the initial titer before treatment (referred to as the control in the *x* axis). Values represent the mean ± standard deviation (*n* = 5).

Considering only the in-storage loss, the three solid formulations maintained the functional titer after 1 week at 21.5 °C (Figure 6). Only the solid formulation S2 presented a functional titer loss of 29.1 ± 42.4% (non-significant, *p* > 0.05).

### 3.3. Intranasal Delivery of the Ad-CMV-F Vaccine Provides Protection in Poultry against a Lethal Challenge with NDV

**Figure 7:**
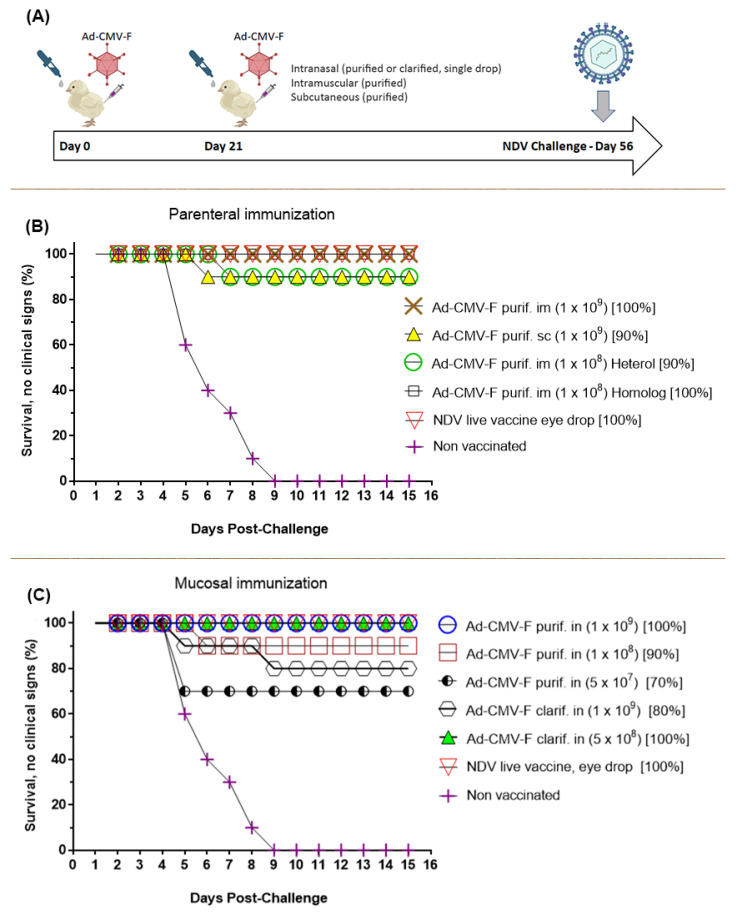
Schematic representation (**A**) and results of the immunization experiment, in which chickens were vaccinated by parenteral (**B**) injection of the CsCl-purified Ad-CMV-F adenovirus or were mucosally immunized (**C**) by intranasal delivery of the virus purified or clarified by filtration steps. All animals were challenged 56 days after the primary immunization with the homologous strain. One of the groups vaccinated by intramuscular injection with purified adenovirus was challenged with both homologous and heterologous NDV strains. The doses evaluated ranged from 1 × 10^9^ to 5 × 10^7^ TCID50 viral particles per animal, and a high degree of protection was seen in all groups. Remarkably, the group i.n immunized with 5 × 10^8^ TCID50 infectious particles per animal reached 100% protection to the NDV challenge. For each animal group, the dose is shown between parentheses and the percentage of protection is indicated.

The production lots were used for the mucosal immunization of chickens via drop instillation of 100 or 200 µL of the vaccine into the nasal cavity. The vaccines were prepared in liquid formulation L2 and delivered on days 0 and 21 of the trial (Figure 7A). The assay evaluated the route (i.m. and s.c., doses from 2 × 10^9^ to 0.5 × 10^7^ TCID50), intranasal delivery, and the two purification methods. One heterologous strain of NDV was also used for the lethal challenge, followed by an observation period of two weeks.

Chickens immunized by intramuscular or subcutaneous injection were protected against NDV. The groups of animals mucosally immunized were all protected to different degrees in accordance with the dose administered. The groups vaccinated with the adenoviral vector highly purified by CsCl showed similar protection levels to those parenterally injected. In total, 100% protection, without clinical disease, was observed at the highest dose evaluated, with 90% protection at lower doses. Remarkably, the group of animals vaccinated with the adenoviral vector subjected to the purification by filtration steps and concentration process, at the lower dose here assayed (5 × 10^8^ TCID50 per animal), showed 100% protection (Figure 7B,C), with no animals showing clinical symptoms of the disease or evidence of typical tissue lesions after euthanasia and necropsy analyses. For clarity and simplicity, the vaccination groups and the results from the challenges are shown in two graphics grouped by the route of immunization employed. The survival curve also considers animals that did not die but showed clinical symptoms of the disease.

## 4. Discussion

The adenovirus Ad-CMV-F-vectored vaccine is a genotype-matched candidate against Newcastle disease virus, based on a non-replicative recombinant variant of the human type 5 adenovirus. It carries the transgene for the Fusion protein of the NDV Ethiopian isolate NDV/Debre zeit/2018 [MN909678], which was assigned to genotype VI after phylogenetic analyses. The candidate vaccine was produced in stirred-tank bioreactors using HEK293SF suspension cells and serum-free media, purified in CsCl gradients, and demonstrated full protection against NDV in poultry immunized by intramuscular vaccination [11]. In this study, we further investigated cost-effective upstream and downstream process alternatives, resulting in the development of more streamlined protocols for production in bioreactors followed by a series of filtration, ultrafiltration, and concentration steps. These procedures, with high recoveries, resulted in a highly immunogenic vaccine formulation inducing protective immunity when mucosally administered to poultry. Additionally, liquid and solid formulations of the adeno-vectored vaccine were developed, showing the remarkable stability of the vaccine when stored under standard conditions for prolonged time or under thermal stressing conditions.

The experiments conducted in shake flasks and the productions in bioreactors showed the suitability of the media evaluated and the fed-batch mode of production to effectively achieve volumetric yields of 1 to 5 × 10^12^ IVP per liter when MOI of 3 is used for cell culture infection at a cell density of around 2–2.5 × 10^6^ cells/mL. Such yields are in the highest range of reports in the literature [26]. This process in bioreactors was previously studied, documented [11], and further optimized and validated by different productions of the Ad-CMV-F adenoviral vector product, as described here. The methods described constitute an effective process for the rapid generation of the raw material to be processed and formulated for mucosal administration. After the adenovirus production, the clarification/purification method was established, using depth filtration and tangential flow filtration, as a scalable and suitable alternative for veterinary vaccines, in which the low cost of production plays a vital role for rapid implementation in the field and commercial success. Even though, to our knowledge, no specific limit of residual host cell DNA appears to be fixed or established to veterinary products, DNase treatment with Benzonase was conducted to successfully reduce the DNA content and DNA length prior to the clarification steps. At the time of ultrafiltration, the hollow-fibre columns evaluated, at a 300 KDa and 500 KDa cut-off, resulted in similar levels of residual host cell protein and residual DNA left after filtration and buffer exchange. The 300 kDa cut-off column was far superior, with high recoveries and negligible virus loss in the permeate fraction.

Regarding the stability studies, five liquid formulations were evaluated under storage conditions at 21.5 °C (room temperature) and 37 °C for 1 and 4 weeks. They were also assessed for stability at 4 °C and −80 °C for a prolonged storage time of 24 weeks. At 37 °C, the L2 formulation maintained the virus activity for 1 week and presented a half-life (t1/2) of around 14 days, similar to a previous report [27] for an Ad5 vector formulated in A195 (containing sucrose as the cryoprotectant and polysorbate-80 as non-ionic surfactant). Even short-term stability at a high temperature, such as 37 °C, is considered important for manufacturing, handling, and distribution of vaccines in less developed areas, such as rural locations and home-based producers’ properties. These ones are the main source of poultry production in sub-Saharan countries [4,5,6]. Moreover, the functional or infectious titer was stable after storage for 16 weeks at 21.5 °C and after 24 weeks at 4 °C. Linear regression analyses of the formulation L2 data indicated that the t1/2 for Ad-CMV-F functional titer loss at 4 °C is 94.7 months, i.e., 7.9 years, a slightly longer half-life than previous findings with the A195 formulation [27]. The overall data support the storage of the vaccine candidate in L2 formulation at 4 °C, being also able to endure significant periods of time without refrigeration. The formulation described in the literature as A438, equivalent to the liquid formulation L3, was evaluated in our study and provided poor stability at 37 °C for 1 week (above 60% functional titer loss). Conversely, a previous study found this formulation suitable for storage of Ad5 at 30 °C for 1 month with a loss of 0.24 log [28]. Nevertheless, in the present study, the formulation L3 was stable at 21.5 °C for 4 weeks, similar to what was reported in 2021 for the chimpanzee adenovirus-vectored vaccines ChAdOx1 and ChAdOx2 [24].

The only liquid formulation presenting functional titer loss after storage at −80 °C for 24 weeks was L1, even though the formulation contained 2% of sucrose, a cryoprotectant agent. L2, also containing 2% sucrose, did not show infectious titer loss. The addition of a non-ionic surfactant in L2 might have helped the preservation of the virus. Formulations L4 and L5, containing other cryoprotectants, such as trehalose and sorbitol, were able to preserve the virus at −80 °C for 24 weeks and after one freeze/thaw cycle. The addition of non-ionic surfactants, such as poloxamer 188 and polysorbate-80 in L2 and L4, had a clear positive impact on the stability at 21.5 °C after 4 weeks of storage, compared to formulation L1. Non-ionic surfactants are used in formulation to prevent adsorption and aggregation. The highest preservation in titer at 37 °C after 4 weeks of storage was achieved with formulation L2, containing poloxamer 188 instead of polysorbate-80. In 2014, an investigation on surfactant’s mechanisms of action demonstrated that poloxamer 188 inhibits protein adsorption by forming protein–surfactant complexes of low adsorption affinity [29]. On the contrary, polysorbate-80 does not form protein–surfactant complexes; this non-ionic surfactant inhibits protein adsorption by their preferential location at an interface to which they show sufficient affinity. Also, at low concentrations, it has been reported that poloxamer 188 at 40 °C was stable for up to 6 months in certain buffer conditions [30,31]. The different mechanisms of action of the two non-ionic surfactants evaluated and their stability at high temperatures might have influenced the preservation of the virus differently.

With regards to the lyophilized vaccine, it has been reported that the combination of mannitol and sucrose or inulin has a positive impact on the pH stabilization of Tris buffers during freezing [25]. In the same line, trehalose is another cryoprotectant agent that can prevent pH shifts upon the freezing process of lyophilized viral vectors [32]. The parameters for the lyophilization process were optimized starting from those already reported [24,25]. No in-process functional titer loss was identified for solid formulation S1, which is a noteworthy improvement compared to previous findings [25]. Nonetheless, we noticed a higher in-process loss (above 55%) with formulation S3, which was still lower than the in-process loss reported for ChAdOx1 and ChAdOx2 in 2021 [24] with the same solid formulation.

Notably, in-storage functional titer loss after 1 week at 21.5 °C in the three formulations was non-significant. The stability of the lyophilized formulations at 21.5 °C may enable field distribution under such conditions. Also importantly, the infectious titer was maintained after 24 weeks at 4 °C. Overall, the liquid formulations could maintain the infectious adenoviral titer for at least 4 weeks at 21.5 °C and for 24 weeks at 4 °C. Specifically, we focused on the formulation L2, which maintains functional titers for at least 16 weeks at 21.5 °C and has a half-life of 2 weeks at 37 °C. We also described an optimized cycle for lyophilizing Ad-CMV-F without in-process loss of functional titer and three solid formulations capable of maintaining Ad5 stability at 4 °C for at least 24 weeks. The liquid formulation is seen as the most advantageous because it has satisfactory stability without the need for an additional lyophilization process. In addition, administration does not require reconstitution of the vaccine, and it can be distributed ready to use.

As per the immunogenicity, protective efficacy, and route of administration, we have demonstrated for the first time that a dose as low as 5 × 10^8^ TCID50/mL administered by drop instillation into the animal’s nostril, with a second dose after 21 days, can provide complete protection against a lethal challenge of NDV. The protective capacity of intramuscular immunization had already been demonstrated. Now, we have corroborated that even doses as low as 5 × 10^7^ can protect 70% of the animals, with the particularity that all the animals survived, although three of them presented clinical signs of the disease. The complete protection provided by immunization through the nasal cavity at a dose of 5 × 10^8^ TCID50 supports recent vaccination studies in animal and human populations showing that the nasal route is the most straightforward and suitable mucosal delivery route for vaccine administration as compared to oral, pulmonary, rectal, or vaginal mucosal delivery routes [33]. The nasal-associated lymphoid tissue (NALT) is the primary inductive site for mucosal immunity in the nasopharyngeal tract [34]. In poultry, the NALT is located on the bottom of the nasal septum and both sides of the choanal cleft. Abundant lymphocytes appear to be distributed under the mucosal epithelium of the inferior nasal meatus. There are also diffuse lymphoid tissues under the epithelium of the concha nasalis media and the nasal cavity walls [35]. Recently, protection against genotype VII of the NDV was achieved by intranasal subunit vaccination based on bacterium-like particles bearing the F or HN antigen [36]. This mucosal subunit vaccine is based on a bacterium-like particle (BLP) delivery platform derived from Lactococcus lactis. The NDV antigens F or HN were fused to a protein anchor that was expressed by a recombinant baculovirus and loaded on the surface of BLPs. The vaccine provided as high as 90% protection rate against an intranasal NDV challenge.

For human pathogens, adenovirus-vectored trivalent COVID-19 vaccines expressing the spike, nucleocapsid, and RdRp antigens used in single-dose intranasal immunization regimens have been recently evaluated in murine models [37]. In particular, the chimpanzee Ad-vectored COVID-19 vaccine intranasally delivered was superior to intramuscular immunization in the induction of the tripartite protective immunity, consisting of local and systemic antibody responses, mucosal tissue-resident memory T cells, and mucosal trained innate immunity. Intranasal immunization provided protection against the ancestral SARS-CoV-2 strain and two more recent variants (VOC, B.1.1.7 and B.1.351), showing that mucosal vaccination with adenovirus-based vaccines is an effective vaccination strategy, with capacity to induce an extensive mucosal immunity. Other anti-COVID-19 vaccines based on human adenoviruses advanced to clinical trials and received approval for administration via intranasal delivery in different countries [38]. They are expected to prevent milder cases of illness and block transmission to other people by prompting immune responses when SARS-CoV-2 first enters the body. Other vaccines based on adenoviral vectors from different species or human serotypes were developed against SARS-CoV-2 and were extensively used worldwide, with an exceptional contribution to saving millions of lives during the COVID-19 pandemic [12].

## 5. Conclusions

To prevent NDV infection in poultry and as an alternative to the current egg-based vaccines, we developed, produced, and validated the Ad-CMV-F adenoviral-vectored vaccine. The vaccine is produced using HEK293SF suspension cells cultured in serum-free media in stirred-tank bioreactors. At least two media commercially available can be used in the manufacturing process, yielding, after clarification and sterile filtration, volumetric yields over 10^12^ IVP per liter of culture. The clarification process is also streamlined and does not involve costly chromatography steps, rendering a purified material suitable for veterinary mucosal vaccination. Altogether, including the remarkable stability shown by one of the liquid formulations prepared, this vaccine product and the associated production process becomes a strong candidate for technology transfer and implementation on the manufacturing site of the National Veterinary Institute, Ethiopia, one of the partners in the project. The adenoviral production platform in stirred-tank bioreactors remains an extremely valuable technology for the rapid development and equitable delivery of vaccines against emerging or re-emerging pathogens affecting animal species for a One Health strategy.

## Figures and Tables

**Table 1:** Composition of the five liquid formulations assessed for stability of the Ad-CMV-F adenoviral vector.

Liquid Formulations				
Formulation L1	Formulation L2	Formulation L3	Formulation L4	Formulation L5
100 mM NaCl	25 mM NaCl	35 mM NaCl	100 mM NaCl	25 mM NaCl
10 mM Tris	20 mM Tris	10 mM Histidine	10 mM Tris	10 mM Tris
2 mM MgCl_2_	2 mM MgCl_2_	1 mM MgCl_2_	2 mM MgCl_2_	2 mM MgCl_2_
2% Sucrose	2% Sucrose	7.5% Sucrose	5% Trehalose	5% Trehalose
	0.005% *w*/*v* Poloxamer 188	0.1% *w*/*v* Polysorbate-80	0.5% *v*/*v* Polysorbate-80	0.5% *v*/*v* Polysorbate-80
		0.1 mM EDTA		3% Sorbitol
		0.5% Ethanol		
pH 7.5	pH 7.5	pH 6.6	pH 7.5	pH 7.5

**Table 2:** Composition of the three solid formulations assessed for the stability of the Ad-CMV-F adenoviral vector.

Solid Formulations		
Formulation S1	Formulation S2	Formulation S3
100 mM NaCl	100 mM NaCl	100 mM NaCl
10 mM Tris	10 mM Tris	10 mM Tris
1 mM MgCl_2_	1 mM MgCl_2_	1 mM MgCl_2_
5% Mannitol	5% Mannitol	5% Mannitol
5% Sucrose	5% Trehalose	5% Inulin
pH 8.2	pH 8.2	pH 8.2

**Table 3:** Evaluation in shake flask experiments of two different culture media and a streamlined fed-batch cultivation mode for the production of the Ad-CMV-F adenoviral vector. Infectious titers of ~10^9^ TCID50/mL were measured in culture supernatant of lysed cells. The mean titers of duplicate measurements plus the standard deviations are shown.

Medium	Approach	Supplement	TCID50/mL
			Harvest (mean ± SD)
HyCell Trans-Fx-H #1	No additives		2.78 × 10^8^ ± 2.90 × 10^7^
HyCell Trans-Fx-H #2	Supplementing during the cell growth phase only	Cell Boost 5 bolus at 5% (*v*/*v*)	9.77 × 10^8^ ± 8.53 × 10^7^
HyCell Trans-Fx-H #3	Supplementing during the complete process	Cell Boost 5 bolus at 5% (*v*/*v*)	2.11 × 10^9^ ± 1.49 × 10^8^
HEK-GM #4	No additives		3.82 × 10^8^ ± 1.20 × 10^7^
HEK-GM #5	Supplementing during the cell growth phase only	HEK-FS bolus at 3% (*v*/*v*)	7.22 × 10^8^ ± 5.80 × 10^7^
HEK-GM #6	Supplementing during the complete process	HEK-FS bolus at 3% (*v*/*v*)	1.83 × 10^9^ ± 1.77 × 10^8^

**Table 4:** Different bioreactor production runs were conducted, and the infectious titers shown were calculated in Tissue Culture Infectious Dose 50 assays, before any concentration or purification steps.

Production	MOI	Titer before Purification Steps (TCID50/mL)
Ad-CMV-F Production 1	0.05–1	5.62 × 10^8^
Ad-CMV-F Production 2	1	3.89 × 10^8^
Ad-CMV-F Production 3	3.5	2.01 × 10^9^
Ad-CMV-F Production 4	3.5	5.10 × 10^9^

**Table 5:** Adenoviral titers and step recovery values from fractions before and after the depth filtration and tangential flow filtration steps. The samples from before and after these steps were quantified by TCID50 and ddPCR assays. Different bioreactor runs and faction volumes were used in the experiment.

Test 1	Fraction Volume (mL)	Titer (IVP/mL)	IVP Total	Step Recovery (%)
Harvest	100	5.60 × 10^9^	5.60 × 10^11^	
After Depth Filter + 0.22 µm	200	3.10 × 10^9^	6.20 × 10^11^	100
**Test 2**				
Harvest	875	3.70 × 10^8^	3.24 × 10^11^	
After Depth Filter + 0.22 µm	875	5.60 × 10^8^	4.90 × 10^11^	100
**Test 3**				
Before TFF	480	4.64 × 10^8^	2.22 × 10^11^	
After TFF (300 kDa)	70	4.64 × 10^9^	3.24 × 10^11^	100
**Test 4**				
Before TFF	875	5.60 × 10^8^	4.90 × 10^11^	
After TFF (300 kDa)	190	3.53 × 10^9^	6.71 × 10^11^	100

**Table 6:** Adenoviral titers at different time points of the process are shown for the two production lots used for animal experiments. Analytical quantifications by TCID50 and ddPCR assays were run for the samples under study.

Production	Infectious Titer (TCID50/mL) in Supernatant	Downstream Processing	Starting Volume (mL)	Final Volume after DSP (mL)	Infectious Titer (TCID50/mL)	Total Particles (VG/mL)
Ad-CMV-F Production 1	5.62 × 10^8^	CsCl-purified, sterile filtration	1050	11.5	1.58 × 10^10^	7.60 × 10^10^
Ad-CMV-F Production 4	3.06 × 10^9^	Depth filtration, TFF, final Amicon concentration, sterile filtration	540	16.5	3.10 × 10^10^	2.71 × 10^10^

**Table 7:** Optimized lyophilization cycle.

Step	Shelf Temp (°C)	Pressure (mTorr)	Time (min)
Loading	5	atm	1
Freezing	−50	atm	55
Freezing	−50	atm	120
Annealing	−15	atm	120
Freezing	−50	atm	120
Vacuum	−50	22	30
1st drying	−50	22	2100
2nd drying ramp	0	22	50
2nd drying	0	22	240
3rd drying ramp	20	22	20
3rd drying	20	22	900

## Data Availability

Most data supporting the conclusions appears in the body of the manuscript. Additional raw data generated in the study are available from the authors upon request.
